# Sleep Worry Among Clinical Nurses: A Cross-Sectional Study

**DOI:** 10.1097/AJN.0000000000000352

**Published:** 2026-07-23

**Authors:** Xiaohan Zhou, Xumin Wang

**Affiliations:** **Xiaohan Zhou** and **Xumin Wang** are both RNs at The First Affiliated Hospital of Soochow University, Suzhou, Jiangsu, China. Contact author: Xumin Wang, jih4q8@sina.com. The authors have disclosed no potential conflicts of interest, financial or otherwise.

**Keywords:** management, nurses, self-care, sleep, sleep anxiety, sleep worry

## Abstract

**Background:**

Sleep-related worry is recognized as a significant determinant of sleep quality. Yet the prevalence and correlates of sleep worry among clinical nurses have not been well established.

**Purpose:**

This cross-sectional study aimed to investigate the prevalence and correlates of sleep worry among clinical nurses in order to guide the development of targeted intervention strategies.

**Methods:**

Clinical nurses employed at a large urban hospital in China from June to November 2024 were recruited for this study. A rigorously developed and validated sleep worry scale was used to assess each nurse's level of sleep worry. Correlational analysis was used to identify significant associations between variables of interest and sleep worry. Multiple linear regression analysis was used to identify significant influencing factors of sleep worry.

**Results:**

A total of 285 clinical nurses were included in the study. Within this cohort, the prevalence of sleep worry was 64%; the mean sleep worry scale score was 32.14. Correlation analysis revealed significant associations between sleep worry and participants' age, work department, professional title, years of nursing experience, and number of night shifts per month. Multiple linear regression analysis identified age, work department, professional title, years of nursing experience, and number of night shifts per month as significant influencing factors of sleep worry.

**Conclusions:**

The high prevalence of sleep worry among clinical nurses underscores the need for targeted and effective intervention strategies. The identified influencing factors provide valuable guidance for the development of these strategies. Such measures are essential to effectively mitigate sleep worry among clinical nurses, thereby potentially enhancing their overall well-being and job performance.

Hospital patient care requires 24/7 coverage, which makes shift work a common practice in nursing. According to one recent survey, in China's tertiary hospitals over 90% of nurses participate in shift work, with the majority working five or more night shifts per week.^1^ The disruptions to circadian rhythms associated with rotating and night shift work, coupled with the high stress levels often associated with nursing work, adversely impact nurses' physiological and psychological well-being and make them more susceptible to sleep-related problems.^2^

In recent years, there has been increased interest in understanding the factors affecting nurses' physical and mental health, with many studies focusing on sleep issues among nurses.^3^ This was particularly evident during the COVID-19 pandemic, when frontline nurses faced immense work pressures and heavy psychological burdens, leading to or exacerbating sleep problems.^4^ Research indicates that sleep disorders increase the risks of illnesses such as cardiovascular disease and diabetes,^5^ as well as of developing or worsening mood disorders such as depression and anxiety.^6^,^7^ Moreover, sleep disorders can impair cognitive functions, including attention, memory, and decision-making, which in turn could impact job performance, potentially lowering care quality and risking patients' safety.^6^,^8^

Sleep worry (also called sleep anxiety) has been defined as “worries about the consequences of poor sleep and the uncontrollability of sleep.”^7^ There is evidence that people with sleep disorders such as insufficient sleep duration, poor sleep quality, and insomnia (persistent difficulty initiating or maintaining sleep) are more likely to experience sleep worry, which in itself can induce depressive symptoms and affective disorders.^7^,^9^ This pattern can become a vicious cycle, since excessive worry about sleep can activate the sympathetic nervous system, leading to presleep cognitive arousal as well as emotional distress and thereby inducing insomnia.^7^,^10^ Yet previous studies exploring sleep among clinical nurses have mainly focused on sleep quality, with little attention to sleep worry.

**Study purpose**. This study aimed to investigate the prevalence and correlates of sleep worry among clinical nurses in order to guide the development of targeted interventions and management strategies.

## METHODS

**Design and setting**. This study employed a cross-sectional survey design. The study site was a tertiary public (government-run) hospital located in Suzhou, Jiangsu Province, China. Ethical approval for the study was granted by the study hospital's ethics committee.

**Sample**. For this study, the researchers recruited clinical nurses working at the hospital from June 1 to November 30, 2024. The inclusion criteria were as follows: having a nursing practice certificate, having at least six months' experience working as a clinical nurse, being scheduled to work both day and night shifts according to the roster in their current department, and being able to understand the research objectives and significance (to that end, the researchers used a standardized set of instructions to explain the study content in detail to prospective participants). Participation was voluntary. Eligible nurses who wished to participate were asked to sign the informed consent form prior to data collection. Nursing interns, visiting nurses, and nurses who did not want to participate were excluded, as were participants who withdrew before completing the survey.

**Data collection**. Regarding participant characteristics, the following information was collected: gender, age, marital status, educational level, work department, professional title, years of nursing experience, employment status, number of night shifts per month, and average monthly income. (The participating nurses followed a fixed rotation system of day and night shifts [for example, two to three day shifts followed by one to two night shifts, with rest days in between], per their department rosters. This system ensures 24-hour nursing coverage and is standard scheduling practice in the study hospital.)

To assess the level of sleep-related worry among nurses, this study employed a rigorously developed and validated survey, the Anxiety and Preoccupation About Sleep Questionnaire.^11^ Originally constructed by British researchers Tang and Harvey in 2004,^11^ the tool was subsequently translated into Chinese, and culturally adapted to ensure its applicability to Chinese nurses, by Shi and colleagues.^12^ The scale consists of 10 items and uses a 5-point Likert-type scale. Response options range from 1 (strongly disagree) to 5 (strongly agree). Total possible scores range from 10 to 50 points, with higher scores indicating a greater degree of sleep worry. A total score of 30 or more points suggests that the nurse has significant sleep worry. In terms of psychometric properties, the scale has demonstrated excellent measurement characteristics. Shi and colleagues found that Cronbach α was 0.88, indicating a high degree of internal consistency; the Spearman-Brown split-half reliability coefficient was 0.92, indicating good reliability; and the test-retest reliability coefficient over a four-week period was 0.73, indicating good temporal stability.^12^

To maintain participants' anonymity, all questionnaires were assigned unique serial numbers instead of personal identifiers. If a participant had any questions during the process of completing the questionnaire, they were encouraged to contact the researchers, who would promptly provide clarification, thus helping to ensure response accuracy. All questionnaires were collected on-site, and the researchers conducted immediate quality checks for completeness. In cases of missing responses or other missing data, the participant was asked to add the necessary information. Completed questionnaires were stored in locked cabinets, and data entry was done using the serial numbers.

**Data analysis**. Statistical analyses were conducted using IBM SPSS version 25 software. Quantitative data that conformed to a normal distribution were presented as means with standard deviations, and intergroup comparisons were performed using either independent samples *t* tests or paired samples *t* tests, as appropriate. Categorical data were expressed as frequencies and percentages and analyzed using the chi-square (χ^2^) test. Pearson or Spearman correlational analyses were employed to assess the associations between participants' personal characteristics and their sleep worry scores. Multiple linear regression analyses were conducted to identify factors that influenced sleep worry in the sample cohort. For all tests, statistical significance was set at *P* < 0.05.

## RESULTS

A total of 285 clinical nurses were included in the study sample. The majority (94%) were female; most (51%) were 30 to 40 years of age. More than half of the participants (61%) had 10 or more years of nursing work experience. Seventy-two percent reported working five or more night shifts per month.

Of the 285 participating nurses, 182 (63.86%) scored 30 or higher on the sleep worry scale, indicating that nearly two-thirds had significant levels of sleep worry. Across the full sample, the mean sleep worry score was 32.14. There were statistically significant differences in participants' sleep worry scores with regard to age, work department, professional title, years of nursing experience, and number of night shifts per month. No significant differences in sleep worry scores were found with regard to gender, marital status, educational level, employment status, and average monthly income. (For detailed participant characteristics and sleep worry scores, see Table 1.)

**Table 1.** Participant Characteristics, with APSQ Results (N = 285)

		APSQ Results		
Variable	No. (%)	Mean Score (SD)	*t/F*	*P* Value^a^
Gender			1.94	0.10
Female	267 (93.68)	32.59 (3.95)		
Male	18 (6.32)	31.02 (4.14)		
Age in years			1.44	0.03
<30	102 (35.79)	35.23 (3.70)		
30-40	144 (50.53)	32.51 (4.03)		
>40	39 (13.68)	30.17 (3.28)		
Marital status			2.25	0.12
Married	210 (73.68)	33.06 (3.22)		
Unmarried	75 (26.32)	31.98 (4.16)		
Educational level			1.34	0.09
Associate degree	32 (11.23)	32.02 (3.85)		
Bachelor's degree	248 (87.02)	32.24 (4.12)		
Master's degree	5 (1.75)	33.01 (3.85)		
Work department			1.00	0.007
Internal medicine	81 (28.42)	32.23 (3.79)		
Surgery	70 (24.56)	31.07 (4.03)		
ICU	56 (19.65)	35.10 (3.55)		
ED	38 (13.33)	33.85 (3.70)		
Pediatrics	20 (7.02)	33.12 (4.28)		
Outpatient	16 (5.61)	30.04 (3.15)		
Other	4 (1.40)	31.12 (3.93)		
Professional title			1.86	0.04
Junior nurse	94 (32.98)	34.58 (3.80)		
NP	118 (41.40)	32.30 (4.37)		
Senior nurse	69 (24.21)	31.04 (3.99)		
Deputy chief nurse	4 (1.40)	30.15 (4.24)		
Years working as a nurse			2.01	0.045
<10	112 (39.30)	33.05 (3.75)		
10-20	150 (52.63)	32.14 (4.04)		
>20	23 (8.07)	30.29 (3.89)		
Employment status			1.43	0.07
Contract-based employment	249 (87.37)	33.18 (4.06)		
Permanent staff	36 (12.63)	31.77 (3.87)		
No. of night shifts per month			1.45	0.02
≤4	81 (28.42)	30.84 (4.25)		
5-7	164 (57.54)	32.90 (4.13)		
≥8	40 (14.04)	34.72 (3.96)		
Average monthly income (in yuan)			1.04	0.11
<5,000	34 (11.93)	33.02 (4.16)		
>5,000	251 (88.07)	32.06 (3.92)		

APSQ = Anxiety and Preoccupation About Sleep Questionnaire.

a Significance was set at *P* < 0.05.

Note: Because of rounding, some percentages may not sum to 100.

Correlation analysis results showed that the nurses' age (*r* = 0.60), work department (*r* = 0.63), professional title (*r* = 0.54), years of nursing experience (*r* = 0.61), and number of night shifts per month (*r* = 0.64) were significantly correlated with sleep worry (see Table 2).

**Table 2.** Correlational Analysis Results: Participant Characteristics and APSQ Scores

Characteristic	*r*	*P* Value^a^
Gender	0.11	0.09
Age	0.60	0.03
Marital status	0.12	0.25
Educational level	0.18	0.08
Work department	0.63	0.02
Professional title	0.54	0.04
Years working as a nurse	0.61	0.04
Employment status	0.25	0.14
No. of night shifts per month	0.64	0.005
Average monthly income (in yuan)	0.16	0.20

APSQ = Anxiety and Preoccupation About Sleep Questionnaire.

a Significance was set at *P* < 0.05.

Multiple linear regression analyses showed that age, work department, professional title, years of nursing experience, and number of night shifts per month were significant influencing factors for sleep worry (see Table 3).

**Table 3.** Multiple Linear Regression Analysis Results: Influencing Factors of APSQ Scores Among Participants

Variable	Partial Regression Coefficient	Standard Error	Standardized Regression Coefficient	*t*	*P* Value^a^
Constant	−26.33	2.14	––	–6.96	0.02
Age	–0.61	0.24	0.42	–1.24	0.02
Work department	0.75	0.15	0.36	3.10	0.02
Professional title	–0.29	0.22	0.38	–2.54	0.04
Years working as a nurse	–0.81	0.12	0.38	–4.76	0.03
No. of night shifts per month	0.86	0.14	0.31	4.43	0.002

APSQ = Anxiety and Preoccupation About Sleep Questionnaire.

a Significance was set at *P* < 0.05.

*R*^2^ = 0.549; adjusted *R*^2^ = 0.505; *F* = 51.86.

## DISCUSSION

When sleep worry activates the sympathetic nervous system, as noted above, the resulting state of arousal can in turn heighten somatic and psychological distress and generate further worry.^13^ In the present study, we found that the incidence of sleep worry among clinical nurses was relatively high. Given the vital role nurses play in patient care, this finding is alarming. Sleep worry can adversely affect not only nurses' physical and mental health, but also their job performance and retention intention.^14^,^15^ Sleep worry can also contribute to poor sleep quality, which has been associated with increased risk of medical errors.^16^,^17^

Clearly, identifying the factors that influence sleep worry in nurses and using that information to develop interventions are critical to the health and safety of both nurses and their patients. We advocate integrating nurses' sleep health into hospitals' comprehensive employee health management systems. Through regular sleep worry assessments and targeted interventions, it may be possible to reduce the incidence and severity of sleep worry in nurses, thereby likely enhancing their overall well-being and, by extension, the quality of patient care. Specific suggestions for action based on this study's findings follow.

Multiple linear regression analyses revealed five factors that significantly influence sleep worry. First, younger age increased the likelihood of sleep worry. This finding may have several underlying reasons. In general, younger nurses will be at earlier stages in their careers; as such, they may lack sufficient work experience and be less resilient in the face of work stressors,^14^ making them more vulnerable to sleep worry. Younger nurses might also be more inclined to take on the unique challenges associated with working nights and thus unintentionally trigger or exacerbate sleep problems. Nurse managers should pay particular attention to younger nurses' mental well-being and ability to handle their workloads, and provide necessary support and training. For example, a mentorship program in which experienced senior nurses offer psychological support and career guidance could help younger nurses better adapt to the work environment. Hospitals could regularly present mental health seminars and provide stress management training sessions aimed at equipping younger nurses with the skills to cope with work pressure.

Regarding work department, participants working in the ICU or ED were more likely to experience sleep worry than those working in other departments. This finding is likely related to the nature of work in the ICU and ED, where nurses generally deal with higher patient acuity, a faster pace of work, and more frequent emergencies, which places them in a state of prolonged high stress.^18^,^19^ This continuous high-pressure environment could further disrupt the circadian rhythms of nurses working rotating or night shifts,^4^ thereby worsening their risk of sleep worry. Targeted psychological interventions and scheduling strategies should be developed for ICU and ED nurses. For example, flexible scheduling could be introduced as a means of adjusting shifts based on individual nurses' needs and fatigue levels. Hospitals could also provide regular psychological support services for nurses. Although not specific to sleep worry, a relevant meta-analysis by Musker and Othman found that offering nurses mindfulness-based interventions effectively helped to alleviate their work-related stress and mitigate burnout.^20^

In our study, participants with hierarchically lower professional titles were more likely to experience sleep worry than those with higher titles. This finding might be associated with the nature of their role on the care team, as nurses with lower titles often undertake the more basic and repetitive tasks and have limited decision-making authority. Compared to nurses with higher titles, those with lower titles may feel more pressure to advance their careers; they might also lack sufficient resources and support to address work-related challenges.^21^ Nurse managers should consider the professional development needs of nurses with lower titles, and support more training and offer guidance for career advancement. Similarly, hospitals should establish a clear career development pathway that provides these nurses with accessible skills training programs and promotional opportunities. Moreover, encouraging nurses with lower titles to participate in departmental management and decision-making processes can enhance their sense of belonging and achievement,^22^ which in turn could reduce their risk of sleep worry.

Participants with shorter nursing tenures and those who worked a greater number of night shifts were more susceptible to sleep worry. The former finding could reflect the possibility that nurses with limited work experience haven't yet fully adapted to the pace and stress of nursing. And given that night shift work is known to disrupt circadian rhythms and interfere with sleep quality,^23^ it stands to reason that working more night shifts would also increase the likelihood of sleep worry. Therefore, nurse managers should optimize scheduling systems to minimize night shift frequency and provide rotating-shift and night shift nurses with more opportunities for rest and recovery. For instance, Lin and colleagues recommend replacing “rapid” shift rotation schedules (alternating day and night shifts within a short time, such as a week) with flexible scheduling and longer shift rotation cycles in order to reduce the impact of night shifts on nurses' circadian rhythms.^24^ Hospitals could provide dedicated rest areas and make nutritional food options available at night, to better support night shift nurses' well-being.

Along with the organizational- and nurse manager–level interventions outlined above, individual-level strategies merit equal attention. These should be tailored to the key influencing factors identified by this study in order to empower nurses to proactively address sleep worry. For younger nurses and those with shorter tenures, engaging in evidence-based stress management techniques (such as mindfulness training^20^) can reduce work-related rumination and presleep arousal. Similarly, seeking mentorship from more experienced nurses may help newer nurses to adapt to the demands of the job and ease career uncertainty. For nurses working in high-acuity settings like the ICU or ED––departments associated with greater sleep worry––using presleep relaxation techniques (such as diaphragmatic breathing) can lessen presleep arousal and, if used consistently, help to break the stress–sleep worry cycle. When additional skills trainings or career development programs are available, nurses with lower professional titles should actively participate as a way to build clinical competence and better understand promotional pathways, which again may ease the stress–sleep worry cycle. Lastly, nurses who work rotating or night shifts can advocate for, and take advantage of, flexible scheduling and meal and rest breaks at their institution. (For more on supporting night shift nurses, see “Shining a Light on the Challenges of Night Shift Nursing: A Mixed-Methods Study” in *AJN*'s July 2026 issue.)

**Limitations and further research**. This study has certain limitations that should be considered in the interpretation and application of the results. First, the study's single-site, cross-sectional design limited the sample size, which restricts the generalizability of the findings. Additional studies using multisite settings and larger sample sizes are recommended. Second, there may be other potentially influential factors that this study did not assess, such as individual psychological resilience, family support systems, and workplace culture. Future studies should expand the range of variables of interest and adopt a more comprehensive analytical framework in order to more thoroughly investigate the multidimensional factors influencing sleep worry among nurses. Third, as this study was cross-sectional in nature, it could not identify causal relationships between variables. Longitudinal or interventional studies should be conducted to elucidate the dynamic mechanisms by which various factors influence sleep worry among nurses.

Future research should also delve into possible associations between sleep worry and nurses' job performance and patient safety. Explorations into how innovative strategies and multidisciplinary collaboration might impact nurses' sleep quality and overall health are warranted. For instance, a study could use artificial intelligence–based sleep monitoring to gather real-life data on nurses' sleep patterns, then investigate how including that data in big data analysis impacts personalized intervention plans. Hospital management could encourage nursing collaboration with experts in fields such as psychology and sleep medicine in order to develop multidimensional and multilevel health management models for nurses that better address sleep worry and its deleterious effects. Taken together, this additional research will provide a more reliable evidence base for the development of scientifically sound and effective sleep–worry intervention strategies.

## CONCLUSIONS

This study revealed a high prevalence of sleep worry among clinical nurses and identified five influencing factors: age, professional title, work department, years of nursing experience, and number of night shifts per month. For nurses who are younger, hold lower professional titles, work in the ICU or ED, have shorter nursing tenures, or work a greater number of night shifts, targeted interventions to alleviate sleep worry are essential and recommended. These should include psychological support, additional skills training and clear career advancement pathways, and optimized scheduling. This study's findings offer valuable guidance for the development of targeted and effective intervention strategies as well as future research.
